# Sociobiome - Individual and neighborhood socioeconomic status influence the gut microbiome in a multi-ethnic population in the US

**DOI:** 10.21203/rs.3.rs-2733916/v1

**Published:** 2023-04-19

**Authors:** Jiyoung Ahn, Soyoung Kwak, Mykhaylo Usyk, Dia Beggs, Heesun Choi, Dariush Ahdoot, Feng Wu, Lorraine Maceda, Huilin Li, Eun-Ok Im, Hae-Ra Han, Eunjung Lee, Anna Wu, Richard Hayes

**Affiliations:** NYU Langone Health; NYU Grossman School of Medicine; NYU Grossman School of Medicine; Department of Population Health, NYU Grossman School of Medicine; NYU Grossman School of Medicine; NYU Grossman School of Medicine; Perlmutter Cancer Center, NYU Langone Health; NYU Grossman School of Medicine; NYU Grossman School of Medicine; Emory University; Johns Hopkins University School of Nursing; University of Southern California; University of Southern California; Department of Population Health, NYU Grossman School of Medicine

**Keywords:** Socioeconomic status, SES, gut microbiome, social epidemiology, population-based study

## Abstract

Lower socioeconomic status (SES) is related to increased incidence and mortality due to chronic diseases in adults. Association between SES variables and gut microbiome variation has been observed in adults at the population level, suggesting that biological mechanisms may underlie the SES associations; however, there is a need for larger U.S. studies that consider individual- and neighborhood-level measures of SES in racially diverse populations. In 825 participants from a multi-ethnic cohort, we investigated how SES shapes the gut microbiome. We determined the relationship of a range of several individual- and neighborhood-level SES indicators with the gut microbiome. Individual education level and occupation were self-reported by questionnaire. Geocoding was applied to link participants’ addresses with neighborhood census tract socioeconomic indicators, including average income and social deprivation in the census tract. Gut microbiome was measured using 16SV4 region rRNA gene sequencing of stool samples. We compared α-diversity, β-diversity, and taxonomic and functional pathway abundance by socioeconomic status. Lower SES was significantly associated with greater α-diversity and compositional differences among groups, as measured by β-diversity. Several taxa related to low SES were identified, especially an increasing abundance of Genus *Catenibacterium* and *Prevotella copri*. The significant association between SES and gut microbiota remained even after considering the race/ethnicity in this racially diverse cohort. Together, these results showed that lower socioeconomic status was strongly associated with compositional and taxonomic measures of the gut microbiome, suggesting that SES may shape the gut microbiota.

## Introduction

Lower socioeconomic status (SES) is related to increased incidence and mortality due to chronic diseases, including cancer, cardiovascular disease, and diabetes (1–3). While socioeconomic inequalities in health are well-established, the biological mechanisms that underlie SES-related health disparities are less well understood. Low SES is associated with multiple health-related behaviors, such as reduced access to medical and dental care (4), increased engagement in unhealthy behaviors such as smoking and alcohol dependency (5), and decreased engagement in positive health behaviors such as healthy eating and exercise (6). We proposed that the gut microbiome may mediate the relationship between SES and chronic disease, because of growing evidence showing that the gut microbiome is often impacted by these same factors (7–9).

The gut microbiome is largely established by the fourth year of life (10) and there is abundant evidence that maternal and family SES influence the infant and childhood gut microbiome (11–17). An important indicator that SES might also influence the gut microbiota in adulthood comes from studies showing that twin pairs who experience a different SES in adulthood also tend to exhibit a differential gut microbiome (18); furthermore, multi-generational studies indicate that heritability plays only a minor role in gut composition of family members (19). Recent studies of the gut microbiome in adults from the U.S. (20), China (21), and the U.K. (18) also point to SES-related gut microbiome differentials at the population level; however, these studies were limited by small sample size (n = 44) (20), limited microbial characterization (21), or study population homogeneity (18). Therefore, there is a need for larger studies that consider individual and area-related measures of SES in racially diverse populations (9, 22).

In a study of 825 participants of diverse race and nativity in the United States, in the Food and Microbiome Longitudinal Investigation (FAMiLI) study, we investigated whether low SES, assessed by individual and neighborhood characteristics, is associated with overall gut microbiota diversity and composition and with specific microbial taxon abundances. We also explored if the SES and the gut microbiome association differs by race/ethnicity. As the FAMiLI study was specifically designed to include diverse populations by race and nativity, we had the unique opportunity to evaluate SES—microbiome relationships in the context of a widely diverse population.

## Subjects And Methods

### Study population

Detailed information on the FAMiLI study population is available elsewhere (23). Briefly, FAMiLI is an ongoing multi-ethnic prospective study in the United States, initiated in 2016. Participants aged 40 years or over were recruited, completed demographic and dietary questionnaires, and provided stool samples. For the current analysis, we used previously sequenced stool samples from 873 participants recruited between 2016 and 2018 with available SES and demographic data and who did not use antibiotics in the 2 weeks prior to the stool collection (23). Participants with missing or unknown data on age, gender, race/ethnicity or nativity (n = 10) were excluded from analysis. We further excluded those whose sequencing failed (n = 9), and subjects with insufficient stool sample gut microbial richness (n = 29), resulting in the final sample size of 825 subjects from varying racial/ethnic (White, Asian, Black, and Hispanic), and nativity (U.S.-born and foreign-born) backgrounds (Table 1).

### Socioeconomic Status

Socioeconomic status is an individual’s relative social and economic position in relation to others (24, 25). SES is often characterized for individuals by measures of education, occupation, and/or income (25). SES can also be conceptualized and measured at the neighborhood-level, that is where a person lives. Neighborhood-level SES may serve as a proxy for individual-level SES (26), but may also be considered as a separate environmental SES indicator, which may influence health outcomes independently of individual SES (27, 28). Herein, we examined two individual-level (education and occupational socioeconomic index) and two neighborhood-level SES indicators (neighborhood income and social deprivation index) that represent socioeconomic status across these two domains.

Individual education and occupation were self-reported by questionnaire. The education level was classified as either a) high school graduate or less or b) more than high school. Participant’s self-reported usual occupation was matched to a corresponding standard occupational classification and U.S. Census Bureau’s coding scheme, and then assigned the occupational socioeconomic index (OSEI), which reflects the education, income, and prestige associated with an individual’s occupation (29). The OSEI score was not assigned to those who did not provide their occupation or were not classified in the Census Bureau coding scheme (i.e., homemakers, unemployed, others) (15.9%). The OSEI score ranges from 0–100, with lower values indicating greater deprivation.

The area-level SES indicators were derived from the Census Bureau’s American Community Survey (ACS) 5-year summary file (https://www.census.gov/programs-surveys/acs/). Participants’ addresses were geocoded using ArgGIS software (ESRI Inc, Redlands, CA) and coordinates were converted into census tract identifiers. The neighborhood income level was derived using median household income in the census tract (B19013_011: median household income in the past 12 months) obtained from ACS data for 2011–2015. The neighborhood social deprivation index (SDI), a well-validated index of SES (30), is a composite measure of seven neighborhood SES characteristics from ACS data for the years 2011–2015: poverty, education, non-employment, living in a renter-occupied home, living in crowded housing, single-parent household, no car ownership. The SDI ranges from 0–100, with lower values indicating higher deprivation. For the analysis, SDI score was reversed to be ordered by low to high level of SES. The OSEI, neighborhood income, and reversed SDI were categorized into quintiles and were ordered from greatest (Q5) to least (Q1) to estimate the effect of lower SES on gut microbiome profiles.

### Microbiome Assessment

Stool samples underwent 16S rRNA gene sequencing at the Environmental Sample Preparation and Sequencing Facility at Argonne National Laboratory (23, 31). DNA was extracted using the PowerSoil DNA isolation kit (MO BIO Laboratories; Carlsbad, CA), following the manufacturer’s protocol. DNA was amplified for the V4 region of the 16S rRNA gene using the 515F/806R primer pair, which included Illumina flow cell adapter sequences with sample-specific barcodes (32). Sequencing reads were demultiplexed and paired-end reads were joined, followed by quality filtering using the QIIME2 pipeline (33). Next, the Deblur workflow was applied, which uses sequence error profiles to obtain putative error-free sequences, referred to as “sub” operational taxonomic units (s-OTU) (34). The s-OTUs were assigned taxonomy using a naive Bayes classifier pre-trained on the Greengenes 13.8 99% OTUs (35), where the sequences have been trimmed to only include 250 bases from the 16S V4 region, bound by the 515F/806R primer pair. A phylogenetic tree was constructed via sequence alignment with MAFFT (36), filtering the alignment, and applying FastTree (37) to generate the tree.

### Statistical analysis

Gut microbiome profiles characterized by α-diversity, β-diversity, and differential abundance of species were analyzed with respect to individual-level (education and occupational socioeconomic index) and neighborhood-level SES indicators (neighborhood income and social deprivation index), as well as nativity, and race/ethnicity. To calculate α- and β-diversity measures, count data was aggregated to the species level and rarefied to even sampling depth. We measured α-diversity as the observed number of species (species richness, the number of different species in a sample representing the richness of the sample), Shannon diversity index (richness + evenness, representing the richness of sample weighted by the abundance of each of the species), and Faith’s phylogenetic diversity (PD, representing phylogenetic richness considering the similarity between bacteria based on shared evolution (38)). Alpha-diversity measures were calculated using ‘phyloseq’ package (39), and were compared by sociodemographic indicators using linear regression, t-test or one-way ANOVA as appropriate for the respective measures. We measured β-diversity using the Jensen-Shannon Divergence (JSD) distance (40). Beta-diversity measures were calculated using the ‘vegan’ (41) and ‘phyloseq’ packages, and were compared using principal coordinates analysis (PCoA) and permutational multivariate analysis of variance (PERMANOVA) (42). The box plot of the JSD distance and pairwise PERMANOVA were used to, respectively, represent and test the significance of differences between groups. Multivariate PERMANOVA including age, sex, and individual-, and neighborhood-level SES indicators were constructed to compare the explained variance of each of the variables. We assessed correlation between sociodemographic indicators using Cramer’s V, a correlation coefficient for categorical variables (43).

For differential abundance analysis with respect to sociodemographic indicators, analysis of compositions of microbiomes was carried out with bias correction (ANCOM-BC) (44), adjusting for age and sex. Additional ANCOM-BC adjusting for age, sex, and race/ethnicity was conducted to identify SES-related species controlling for race/ethnicity. We used a minimum prevalence filter of 10% and a false discovery rate (FDR) threshold of 0.05 when identifying significantly differentially abundant species. Functional pathways were imputed from 16SV4 region rRNA gene-based microbial compositions using the PICRUSt2 algorithm (45), with reference to the MetaCyc pathway catalog (46). A total of 391 MetaCyc pathways were imputed. Functional pathways relating to the SES indicators were identified by ANCOM-BC, controlling for age and sex. The effect size of the ANCOM-BC identified imputed pathways were visualized in volcano plots and heatmaps. All analyses were conducted using R (4.1.0).

## Results

### Study participants

The current analysis included 825 adults (36.7% male), with a mean age of 59.6 years (Table 1). The racial and ethnic group composition was 311 (37.7%) Whites, 287 (34.8%) Asians, 89 (10.8%) Blacks, and 138 (16.7%) Hispanics. Of the participants, 48.1% were foreign-born and 25.0% had education to high school graduation or less. The range and the number of participants in each quintile of OSEI, neighborhood income, and SDI were presented. The two individual-level SES indicators (education and OSEI) were strongly correlated with each other (Cramer’s V = 0.44, p-value < 0.001), as were the two neighborhood-level indices (neighborhood income and SDI) (Cramer’s V = 0.47, p-value < 0.001). Comparing individual-level to neighborhood-level measures showed correlations ranging from 0.16 to 0.39. Individual- and neighborhood-level SES tended to be correlated with nativity and race/ethnicity. Also, nativity and race/ethnicity were strongly correlated with each other (**Supplementary Fig. 1**).

### Socioeconomic Status And Gut Microbiome Overall Diversity

**Figure 1:**
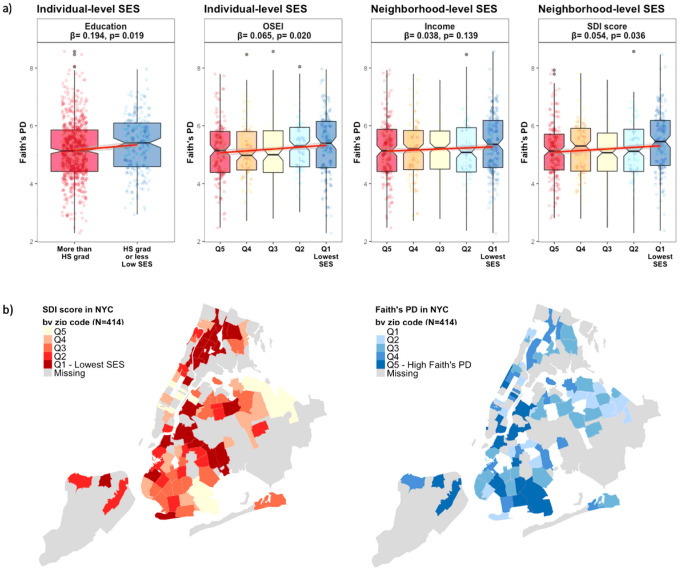
Alpha diversity by socioeconomic characteristics a) Faith’s phylogenetic diversity of 16S rRNA gut microbiome samples. Measures were compared using a null hypothesis of no difference between groups (Regression, p <0.05). b) Visual comparison of SDI score and Faith’s PD in NYC by zip code. Significant positive spatial autocorrelation (Moran’s I= 0.302, p-value= 0.001) was observed for SDI score, and positive but insignificant spatial autocorrelation for Faith’s (Moran’s I= 0.011, p-value= 0.157). Abbreviations: PD: Phylogenetic diversity, HS grad: High School graduate, OSEI: Occupational Socioeconomic Index, SDI: Social Deprivation Index Note. High-resolution figures are provided as a separate attachment behind.

Lower individual educational attainment and occupational status (OSEI) were associated with greater microbial α-diversity represented as the number of phylogenetic tree-units within a sample (Faith’s phylogenetic diversity, p-value 0.02; Fig. 1A). Also, individuals living in areas of greater neighborhood deprivation (SDI score) exhibited greater α-diversity (Faith’s phylogenetic diversity, p-value 0.04, Fig. 1A). Lower educational attainment was associated with Shannon α-diversity, which is sensitive to both richness (total number of species in the community) and evenness (relative abundance of different species (**Supplementary Fig. 2**). As expected, SDI exhibited strong spatial autocorrelation in New York City residents (n = 414, 50.2%) by census tract. Microbiome Faith’s phylogenetic diversity tended also to pattern spatially, however, the spatial autocorrelation was not statistically significant (Fig. 1B).

**Figure 2:**
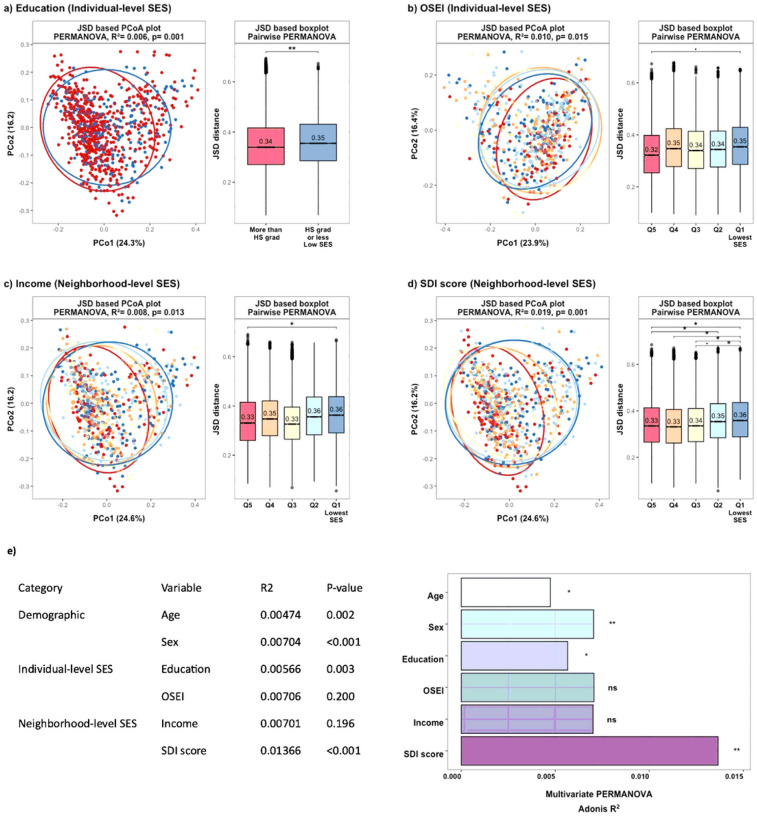
Beta diversity by socioeconomic characteristics a-d) Principal Coordinate Analysis (PCoA) plot and box plot of the JSD distance. Statistical significance between socioeconomic indicators was determined using permutational multivariate analysis of variance (PERMANOVA). The significance of differences among the groups was tested using pairwise-PERMANOVA. e) Multivariate PERMANOVA model. The bars depict the amount of variance (R^2^) explained by each socioeconomic variable in JSD distance. Size effect and statistical significance were calculated by PERMANOVA including sociodemographic variables in one model. Stars denote the level of significance (Bonferroni post-hoc-tests; • p-value <0.05; * p-value <0.01; ** p-value <0.001). HS grad: High School graduate; OSEI: Occupational Socioeconomic Index, SDI: Social Deprivation Index Note. High-resolution figures are provided as a separate attachment behind.

Consistent with findings for α-diversity, overall composition differentials in gut microbiome (β-diversity) were identified with respect to individual- and neighborhood-level SES indicators, as shown in principal coordinate plots and age and sex adjusted JSD boxplots (Fig. 2A–D, PERMANOVA: p-value < 0.05). In the multivariate PERMANOVA model (Fig. 2E), including multiple correlated SES indicators (**Supplementary Fig. 1**), individual education level (p-value = 0.003) and neighborhood-level SDI score (p-value = 0.001) remained significantly associated with microbiome β-diversity, while SDI score had the largest explanatory power on gut microbiome composition (R^2^ = 0.014) than other SES indicators (R^2^ were 0.006 to 0.007).

### Socioeconomic Status And Differential Gut Microbiome Taxa

**Figure 3:**
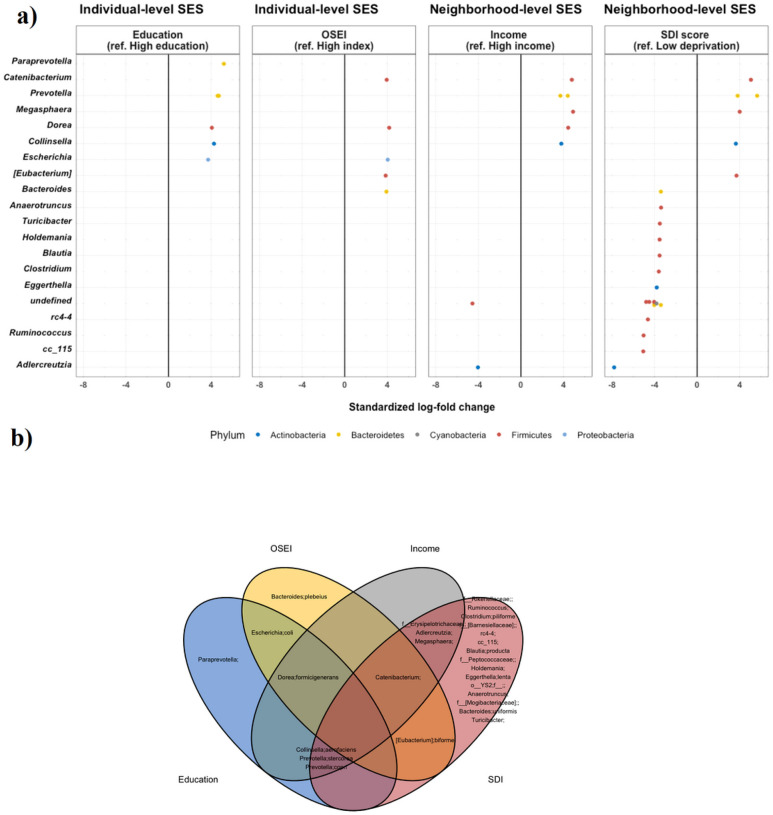
Differential abundance by socioeconomic characteristics a) Plot showing the standardized log-fold changes (x-axis) by genus level (y-axis) derived from the ANCOM-BC model. ANCOM-BC was conducted by each socioeconomic indicator after adjusting for age and sex. Each dot represents a single species and is colored by the phylum level. Standardized log-fold change values greater than 0 indicate the fold change increase in the low SES (deprived) groups, while standardized log-fold change values less than zero indicate the fold change decrease in the low SES groups. b) Venn diagram of shared species by each socioeconomic indicator Note. High-resolution figures are provided as a separate attachment behind.

ANCOM-BC analysis further revealed several gut bacterial species associated with lower SES status (Fig. 3). Twenty-seven species were identified as differentially abundant by SES indicators including 6 species by education, 5 by occupation, 8 by neighborhood income, and 23 by SDI score. SDI score identified the greater number of differential species, and this may be partially explained by the PERMANOVA results, that SDI score had the largest explanatory power on the gut microbiome composition. Lower SES-associated taxa include Genus *Catenibacterium*, *Prevotella copri*, *Prevotella stercorea*, *Dorea formigenerans*, *and Collinsella aerofaciens* (> 4 fold higher; FDR < 0.05) and higher SES-associated taxa include Family *Erysipelotrichaceae* and *Adlercreutzia* (>4 fold lower; FDR < 0.05).

**Figure 4:**
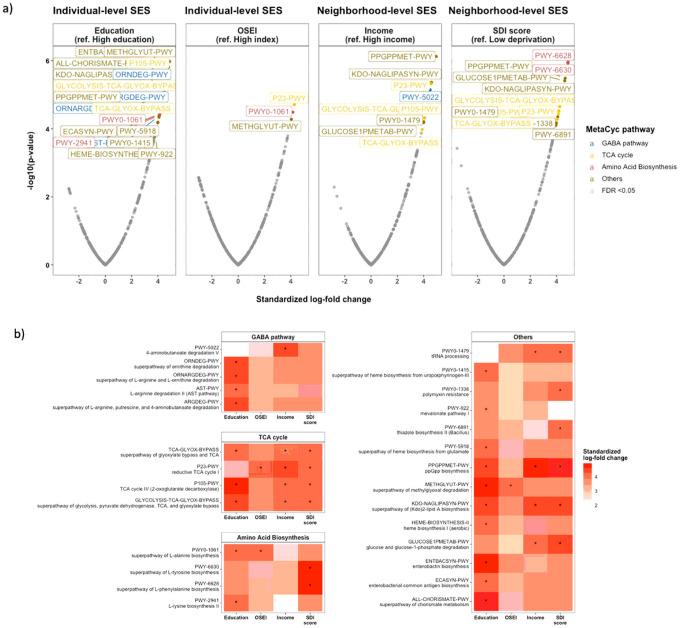
Deprivation of socioeconomic status and functional pathway Functional pathways were predicted from 16S rRNA gene-based microbial compositions using the PICRUSt2 algorithm to make inferences from the MetaCyc pathway database. a) Volcano plot showing the standardized log-fold changes (x-axis) by the negative log-transformed p-value (y-axis) derived from the ANCOM-BC model. ANCOM-BC was conducted by each socioeconomic indicator after adjusting for age and sex. b) Only functional pathways relating to low socioeconomic status are included in the heatmap. Stars denote the significance of the ANCOM-BC (* FDR<0.05). Note. High-resolution figures are provided as a separate attachment behind.

Figure 4 depicts microbiota functional differences across SES status based on imputed pathways using the PICRUSt2 algorithm (Fig. 4). From a total of 391 MetaCyc pathways tested, 27 pathways related to SES were identified by ANCOM-BC after adjusting for age and sex (FDR < 0.05), including 19 pathways by education, 3 by occupation, 9 by neighborhood income, and 12 by SDI score (Fig. 4A). The positive standardized log-fold changes suggested that low SES is related to an increase in certain functional pathways, including the GABA pathway, TCA cycle, and amino acid biosynthesis. Education level and neighborhood income were significantly associated with microbial pathways involved in 4-aminobutanoate (GABA) degradation. Still, other SES indicators (occupation and SDI score) showed similar positive association with GABA pathways (Fig. 4B).

### Effect of race/ethnicity in the relationship between SES and gut microbiota

**Figure 5:**
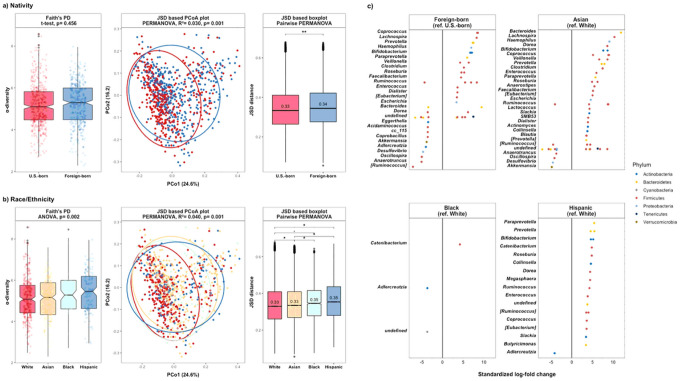
Microbiome profiles by nativity and race/ethnicity a) Nativity α-diversity and β-diversity b) Race/ethnicity α-diversity and β-diversity c) Nativity and race/ethnicity ANCOM-BC. Plot showing the standardized log-fold changes (x-axis) by genus level (y-axis) derived from the ANCOM-BC model. ANCOM-BC was conducted by nativity and race/ethnicity after adjusting for age and sex. Each dot represents a single species and is colored by the phylum level. Standardized log-fold change values greater than 0 indicate the fold change increase in the test group, while standardized log-fold change values less than zero indicate the fold change decrease in the reference groups. Stars denote the level of significance (Bonferroni post-hoc-tests; • p-value <0.05; * p-value <0.01; ** p-value <0.001). Note. High-resolution figures are provided as a separate attachment behind

Blacks and Hispanics had lower SES (i.e., lower education, OSEI, neighborhood income and SDI score) (**Supplementary Table 1**). Foreign-born participants had significantly lower SES than U.S.-born participants. None of the SES indicators had evidence of heterogeneity by race/ethnicity (p-value > 0.05). Adjustment by race/ethnicity or nativity diminished the explained variance captured by SES indicators but did not fully attenuate the significant associations in PERMANOVA models. With respect to nativity and race/ethnicity, the abundance of identified species was similar when comparing Asians to Whites, and foreign-born to U.S.-born (Fig. 5C). Among the differentially abundant species by SDI (Fig. 3), genus *Catenibacterium* (standardized log-fold change = 3.49, FDR = 0.045) and *Prevotella copri* (standardized log-fold change = 3.47, FDR = 0.048) remained statistically significant after adjustment for race/ethnicity.

## Discussion

Our study demonstrated that lower SES was positively associated with gut microbiome diversity and composition. Several bacterial taxa related to low SES were identified, especially among the genus *Catenibacterum* and for *Prevotella copri*. Though high correlations between SES and race/ethnicity were found, stratification and adjustment for race/ethnicity did not account for significant association between low SES and microbiota composition, posing the importance of SES as a determinant of the gut microbiome.

Our study demonstrated that lower SES is related to gut microbiome diversity and microbiome structure. Our findings that, all SES indicators were significantly associated with gut microbiome composition (β-diversity) is consistent with a large twin cohort in the United Kingdom (18). Both studies suggest that gut microbiome β-diversity is moderately associated with both individual and neighborhood-level SES.

Directionality of SES and α-diversity association, however, remains inconsistent. Two previous studies in adults (18, 20) reported low SES is associated with reduced gut α-diversity (47). Unlike our large multi-ethnic cohort, these studies were characterized either by the small sample size (n = 44) (20), or homogenous population with low SES variability (18). In line with our study, other published literature linking SES and gut microbiota in children (11, 14, 16, 17) showed increased α-diversity related to low SES (i.e., comparing divergent socioeconomic schools, villages, area-based deprivation index, and maternal education). The similarity between childhood and adult microbiota is supported by the fact that the microbiota diversity, composition, and maturity tend to stabilize in the fourth years of life (10), remaining so throughout life with further moderate modification by other environmental factors. Low SES has been associated in some settings with poor hygiene and a lack of sanitation which may lead to higher exposure to microorganisms and parasites and to increased α-diversity (48). Our finding that Faith’s phylogenetic diversity showed a particularly robust association with SES may suggest that low SES is associated with a more unique, highly distinct microbial composition than is found in higher SES groups. More research is needed, however, to clarify and understand how SES relates to α-diversity.

We observed similar associations between several taxa and SES, as found in previous studies of adults (18, 21). Twin UK reported increased abundance of genus *Catenibacterium*, in the low neighborhood-level SES groups, similar to our findings (18). In a study from China, the abundance of *Prevotella copri*, *Prevotella stercorea*, *Dorea Formicigenerans*, and *Collinsella aerofaciens* was negatively associated with annual income (21). We additionally compared the abundance of *Bacteroides* and *Prevotella* at genus level, which were noted to be a predictor of body weight (49), and a biomarker for diet and lifestyle (50). We found that low SES indicators were associated with increased abundance of *Prevotella*, and decreased abundance of *Bacteroides*, in line with other studies (20, 21). These differences in the abundance of *Bacteroides* and *Prevotella* may be explained by different dietary habits that are enriched in animal products relative to carbohydrates. The higher abundance of *Prevotella* in low SES has been reported in other studies and explains that higher intake of vegetables and fiber has been associated with (11, 17).

SES-related taxa were also related to nativity and race/ethnicity. Our earlier study revealed significant differences in microbiome composition across nativity and race/ethnicity (23), including finding of differentially abundant *Prevotella copri* and *Catenibacterium*. Higher abundance of *Prevotella copri* was related to Western origin and diet, which is characterized by more consumption of high fiber and low-fat diets than the typical Western diet. The abundance of *Catenibacterium* was related to foreign-born Hispanics. The present work shows that these enriched species are also associated with low SES, especially with the neighborhood SDI score, even after additional adjustment of race/ethnicity.

Recently, the term “sociobiome” has been coined to describe the microbiota composition occurring in residents of a neighborhood or geographic region as a result of similar socioeconomic exposures (8); socioeconomic status, but also broader social context, are of interest. In respect to social equity and health disparity, the socially minoritized populations are more likely to be exposed to environmental conditions that negatively affect health; including limited access to the fresh produce, poor access to the health care services, and poor hygiene. (51). In addition, the built environment and its related environmental exposures related to individual socioeconomic status (income, occupation) may impact the gut microbiota composition, diversity and function (52, 53). Therefore, understanding the sociobiome is warranted, and future studies should consider SES and the broader social context in identifying microbial factors to impact health inequalities.

Our study is the first to investigate the relationship between SES and the gut microbiome in a large racially and ethnically diverse population. The study adds to the body of knowledge on the impact of individual- and neighborhood-level SES on the gut microbiome. Even though the study was relatively large, a limitation remains that the distribution of SES in each race/ethnic group tended to be limited.

In conclusion, our study demonstrated the significant association between SES and gut bacterial profiles across a diverse population. Differentials in SES were associated with α-diversity, β-diversity, the abundance of bacterial species, and microbial functions. Our results support the important role of SES in shaping gut microbiome composition.

## Figures and Tables

**Table 1:** Characteristics of Study Participants

	Overall (N = 825)
**Age**	
Mean (SD)	59.6 (11.1)
**Sex**	
Male	303 (36.7%)
Female	522 (63.3%)
**Nativity**	
U.S.-born	428 (51.9%)
Foreign-born	397 (48.1%)
**Race/Ethnicity**	
White	311 (37.7%)
Asian	287 (34.8%)
Black	89 (10.8%)
Hispanic	138 (16.7%)
**Individual-level SES**	
**Education**	
More than high school graduate	614 (74.4%)
High school graduate or less; Low SES	206 (25.0%)
Missing	5 (0.6%)
**OSEI**	
Q5 [81.025, 92.782]	132 (16.0%)
Q4 [62.947, 80.919]	135 (16.4%)
Q3 [43.859, 62.573]	120 (14.5%)
Q2 [28.681, 42.994]	168 (20.4%)
Q1 [12.609, 28.645]; Lowest SES	139 (16.8%)
Missing	131 (15.9%)
**Neighborhood-level SES**	
**Income**	
Q5 [86302, 209063]	165 (20.0%)
Q4 [63446, 85551]	165 (20.0%)
Q3 [51806, 63036]	165 (20.0%)
Q2 [36250, 51773]	164 (19.9%)
Q1 [11809, 36236]; Lowest SES	166 (20.1%)
**SDI score**	
Q5 [1, 21]	165 (20.0%)
Q4 [22, 48]	170 (20.6%)
Q3 [49, 74]	160 (19.4%)
Q2 [75, 91]	185 (22.4%)
Q1 [92,100]; Lowest SES	145 (17.6%)

Values are presented as the mean (SD) for continuous variables and as the number of counts and percentages for categorical variables. OSEI: Occupational Socioeconomic Index, SDI: Social Deprivation Index

## Data Availability

The 16S rRNA sequencing data that support the findings of this study have been deposited in the Sequence Read Archive (PRJNA559143), along with demographic metadata, to be released upon publication. Additional data on the study participants are available from the corresponding author upon reasonable request.
